# The Effect of miR-4800 Restoration on Proliferation and Migration of Human Breast Cancer Cells *In Vitro*

**DOI:** 10.34172/apb.2023.041

**Published:** 2022-01-05

**Authors:** Monireh Khordadmehr, Reyhaneh Matin, Behzad Baradaran, Elham Baghbani, Farinaz Jigari-Asl, Saeed Noorolyai

**Affiliations:** ^1^Department of Pathobiology, Faculty of Veterinary Medicine, University of Tabriz, 51665-1647, Tabriz, Iran.; ^2^Immunology Research Center, Tabriz University of Medical Sciences, 51666-14761, Tabriz, Iran.; ^3^Department of Immunology, Faculty of Medicine, Tabriz University of Medical Sciences, 51666-14761, Tabriz, Iran.

**Keywords:** microRNA, Malignancy, Replacement, Therapeutic target

## Abstract

***Purpose:*** MicroRNAs (miRNAs) can contribute to cancer initiation, development, and progression. In this study, the effect of miRNA-4800 restoration on the growth and migration inhibition of human breast cancer (BC) cells was investigated.

***Methods:*** For this purpose, transfection of miR-4800 was performed into MDA-MB-231 BC cells using jetPEI. Subsequently, the expression levels of miR-4800 and CXCR4, ROCK1, CD44, and vimentin genes were measured using quantitative real-time polymerase chain reaction (q-RT-PCR) and specific primers. Also, the proliferation inhibition and apoptosis induction of cancer cells were evaluated by MTT and flow cytometry (Annexin V-PI method) techniques, respectively. Additionally, cancer cell migration after miR-4800 transfection was assessed by wound-healing (scratch) assay.

***Results:*** The restoration of miR-4800 in MDA-MB-231 cells resulted in the decreased expression level of CXCR4 (*P* ˂ 0.01), ROCK1 (*P* ˂ 0.0001), CD44 (*P* ˂ 0.0001), and vimentin (*P* ˂ 0.0001) genes. Also, MTT results showed restoration of miR-4800 could significantly reduce cell viability rate (*P* ˂ 0.0001) compared with the control group. Cell migration remarkably inhibited (*P* ˂ 0.001) upon miR-4800 transfection in treated BC cells. Flow cytometry data demonstrated that miR-4800 replacement considerably induced apoptosis in cancer cells (*P* ˂ 0.001) compared with control cells.

***Conclusion:*** Taken together, it seems that miR-4800 can act as a tumor suppressor miRNA in BC and play an essential role in modulating apoptosis, migration, and metastasis in BC. Therefore, it may be suggested as a potential therapeutic target in treating BC by performing additional tests in the future.

## Introduction

 Cancer is the second-largest cause of death worldwide after cardiovascular diseases. Globally, breast cancer (BC) represents the most common cancer in women, which is the second most common cancer after lung cancer.^1^ An estimated 281 550 BC new cases and 44 130 BC deaths (including 43 600 women and 530 men) will occur in 2021 in the United States.^2^ Eighty percent of cancers increase incidence with age in females > 50 years old and is less prevalent in younger people female. However, illness appears to be more aggressive (survival of 5 years Rates: 81% < 45 years; 86% > 65 years).^3^ As expected, it is really happening rarely in men (UK 350 per year) relative to females. In BC, surgery, radiotherapy, chemotherapy, hormone (anti-estrogen) therapy, and/or targeted therapy are called the main therapeutic approaches.^4^ Most morbidity of the BC and mortality is caused by a highly incurable metastatic illness resistant to conventional therapies. Consequently, reducing BC is important to further elucidate the molecular mechanisms for metastasizing BC and creating novelties approaching treatments.^5^ American Cancer Society presented some risk factors related to the formation of BC, such as increasing age, alcohol consumption, obesity, physical inactivity, family genetic history, and pathogenic genetic variations.^2^

 MicroRNAs (miRNAs) have recently been identified effects on different steps of cancer initiation, development, and progression, such as metastasis, epithelial to mesenchymal transition (EMT), invasion, migration, proliferation, angiogenesis, and may prove effective therapeutic targets.^5^ They are a non-coding class of RNA molecules consisting of around 22 long nucleotides, which can promote degradation or repression of target mRNAs translation. Among these, some promote cancer initiation and progression, which call as oncomiRs. Certain miRNAs, in comparison, show tumor-suppressive properties.^6^ In this regard, dysregulation of miR-4800 has been identified in various cancers, such as BC,^7-9^ cervical cancer,^10^ colorectal cancer,^11,12^ esophageal squamous cell carcinoma (ESCC), stomach cancer,^13^ glioma,^14^ liver carcinoma,^15^ and pancreatic cancer. Indeed, downregulation of miR-4800 was investigated in patients with these cancers. However, little information is available on the biological roles of miR-4800-3p in cancer initiation, development, and progression. Therefore, the present study aimed to examine the potential role of miR-4800-3p on the cancer cell proliferation and migration in BC cells *in vivo*, which was studied by quantitative real-time polymerase chain reaction (q-RT-PCR), MTT, flow cytometry, and wound-healing assays.

## Materials and Methods

###  Cell line selection

 In the present study, four BC cell lines (SKBR-3, MDA-MB-468, MDA-MB-231, and MCF-7) were obtained from the Pastor Institute (Tehran, Iran) and maintained standardly based on the institute ҆s recommendation (using RPMI-1640 medium and 10% FBS (Gibco, USA) in an incubator at 37°C with 5% CO2.

 Subsequently, to select one cell line for further experiments, the expression level of miR-4800 was firstly examined in the cell lines by q-RT-PCR. The lower expression level of miR-4800 was observed in the MDA-MB-231, and it was selected for further experiment steps.

###  MicroRNA transfection

 MDA-MB-231 human BC cell line were cultured in a standard cell culture condition as previously described.^16^ Briefly, it is described as following steps:

1. Seeding 2 × 10^5^ cells in a 6‐well plate.
2. Washing the cells by PBS (phosphate‐buffered saline) when the cells reached 50-80% confluently.
3. Transfection of the miR‐4800 mimic using jetPEI reagent (Strasbourg, France).

Moreover, three (24, 48, and 72 h) time intervals and two dosages of 50 and 100 pmol were conducted to find the optimal time and appropriate dosage of transfected miRNA, respectively.^16^ Also, *Caenorhabditis elegans *(Sigma- Aldrich Co.) was transfected as the negative controls miRNA (Table 1).

###  MTT (3 ‐ (4, 5 ‐ dimethylthiazol ‐ 2 ‐ yl) ‐ 2, 5 ‐ diphenyltetrazolium bromide) assay 

 The cell viability upon miR-4800 transfection was studied by MTT test, which briefly described as following steps:

1. Seeding 15 × 10^3^ of miR‐4800 transfected MDA-MD-231 cells into a 96‐well plate.
2. Seeding 15 × 10^3^ of negative control cells into a 96‐well plate.
3. Utilizing MTT solution (2 mg/mL) to incubate the cells at 37℃ for 4 hours.
4. Depleting the cell culture medium from each well.
5. Adding 200 μL of DMSO (dimethyl sulfoxide) plus 25 μL of Sorenson’s buffer to each well incubated for 30 minutes at 37°C (for solubilization of MTT formazan crystals).
6. Evaluation of the absorbance of each well by a microplate reader (Sunrise, Tecan, Switzerland) at 490–570 nm.

###  Reverse Transcriptase-PCR (RT-PCR) and qRT ‐ PCR

 In the present study, the molecular analyses were conducted as follow:

- Isolation of total RNA from the cell pellets (approximately 1 × 10^6^ cells) using RiboEx reagent (Gene All Biotechnology, Seoul, South Korea).
- Determination of the quality and concentration of extracted RNA using a Nano-Drop spectrophotometer (Thermo Fisher Scientific Life Sciences, USA).
- Evaluation of the integrity of the RNA using the agarose gel electrophoresis.
- Synthesis of miR‐4800 (cDNA) using the miRNA Reverse Transcription Kit (Exiqon, Vedbaek, Denmark) according to the manufacturer’s instruction.
- Synthesis of cDNA for the CD44, CXCR4, ROCK1, and vimentin genes using first-strand cDNA synthesis Kit (Thermo, USA) based on the suggested instruction.
- Setting up the temperatures using a thermal cycler (Bio‐ Rad Laboratories, Inc., Hercules, CA).

The q-RT-PCR test was performed using a standard SYBR Green PCR pre-mix (Amplicon, Odense, Denmark) and reaction tubes contained as follow:

- 5 μL of 2X SYBR green premix,
- 0.25 μL of 4 pmol/μL primers (Bioneer, Korea)
- 0.5 μL of relating cDNA
- Up to 10 μL nuclease-free water

In addition, β-actin and U6 were used as a housekeeping gene for mRNA expression analysis and miR-4800 normalization, respectively. Notably, all mentioned reactions were considered in triplicates. The sequences of the primers are presented in Table 1. The cycling was conducted by 94ºC, 59ºC, and 72ºC for 10 seconds, 30 seconds, and 20 seconds, respectively. Finally, the Ct values were analyzed by the 2^−ΔΔCT^ method.

**Table 1:** Primer sequences that were used in the present study

**Primer name**	**Forward / Reverse**	**Sequences**
ROCK1	F	5′‐AATCGTGTGGGATGCTACCT‐3′
	R	5′‐AAAACCCTCAGTGTGTTGTGC‐3'
CXCR4	F	5'-TCTTCCTGCCCACCATCTACTC-3'
	R	5'-TGCAGCCTGTACTTGTCCGTC-3'
CD44	F	5´- CAAGCCACTCCAGGACAAGG-3´
	R	5´- ATCCAAGTGAGGGACTACAACAG-3´
Vimentin	F	5′‐CAGGCAAAGCAGGAGTCCA‐3′
	R	5′‐AAGTTCTCTTCCATTTCACGCA‐3′
β–actin	F	5´- TCCCTGGAGAAGAGCTACG -3´
	R	5´- GTAGTTTCGTGGATGCCACA -3´
U6 snRNA	Target	5´-GCUCGUUCGGCAGCACACAUAUACUAAAAUUGGAACGA
	sequence	ACAGAGAGAAGAUUAGCAUGGCCCCUGCGCAAGGAUGACACGCAAAUUCGUGAAGCGUUCCAUAUUUUU‐3′
Has-miR-4800	Target sequence (stem loop)	5'GTCGTATCCAGTGCAGGGTCCGAGGTATTCGCACTGGATACGACGTGGAC -3'
*C. elegans* miRNA	Target sequence	5'-CGGUACGAUCGCGGCGGGAUAUC-3'

###  Flow cytometry assay for apoptosis evaluation 

 To investigate apoptosis analysis, the Annexin V/propidium iodide method was applied as following steps:

- Seeding the 2 × 10^5^ cells in the 6-well plates.
- Incubation for 24 hours at 37°C.
- Treating the cells (miR-4800 transfection) as previously used and described (after reaching a confluence of 70%).
- Washing the treated cells after 24 hours by PBS.
- Trypsinization of the treated cells by Trypsin/EDTA 2.5%.
- Adding 3 mL complete medium to the cells and moving into the 1.5 ml micro-tubes.
- Centrifugation at 1200 rpm for 5 minutes and aspiration of the supernatant.
- Staining using Annexin V-FITC and Propidium Iodide Double Staining Kit (Invitrogen, USA).
- Final evaluation using FlowJo software on the BD flow cytometry.

###  Wound-healing (scratch) assay 

 The scratch assay was conducted for cell migration examination as following steps:

1. Seeding 10 × 10^4^ MDA-MB-231 cells into 24‐well plates.
2. Incubation for 24 hours at 37°C.
3. Treating the cells (miR-4800 transfection) as previously used and described (after reaching a confluence of 70%).
4. Creating a gap by a sterile sampler tip on the cell surface.
5. Washing the cells by PBS.
6. Observation and computation of cellular migration to the “wound area” at 0, 24, and 48 hours using an inverted microscope (Optika Microscopes, Bergamo, Italy) from multiple microscopic areas.

###  Statistical analysis 

 The present data were analyzed statistically by GraphPad Prism software (GraphPad Prism 4.0, San Diego, CA), Student’s t and ANOVA tests, and a *P* < 0.05 was considered statistically significant.^17^

## Results

###  Cell line selection

**Figure 1:**
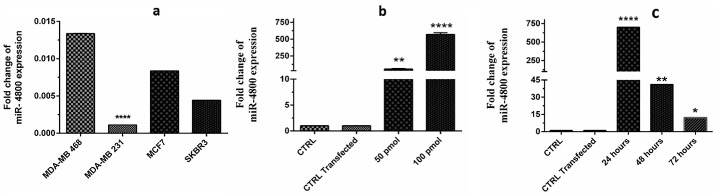
To find the appropriate cell line, effective dosage of transfected miRNA and assessment time, four breast cancer cell lines (a), two dosages of 50 and 100 pmol (b) and three times intervals of 24, 48, 72 h (c) were assessed. The MDA-MB-231 was selected (*P* < 0.0001) for further experiment. Also, the results showed that 100 pmol in the 24 h was the most effective dosage in the most effective time (*P* < 0.0001). The data represent mean ± SD (n = 3), **** *P* < 0.0001.

 The expression level of miR-4800 was evaluated in MDA-MB-468, MDA-MB-231, MCF-7, and SKBR-3 BC cells. The present results showed a remarkable difference between various cell lines (Figure 1a), and finally, the MDA-MB-231 with the lower expression level was selected for further steps (*P*< 0.0001). Also, according to the current findings, 100 pmol (effective dosage) (*P*< 0.0001) of miRNA in 24 hours (effective time) (*P*< 0.0001) was considered to be the most effective adjustment (Figure 1b, c). Here, the miR-4800 mimic transfection resulted in the efficient restoration (88.2%) of miR-4800 in BC cells.

###  Cell viability and migration were decreased after miR-4800 transfection

**Figure 2:**
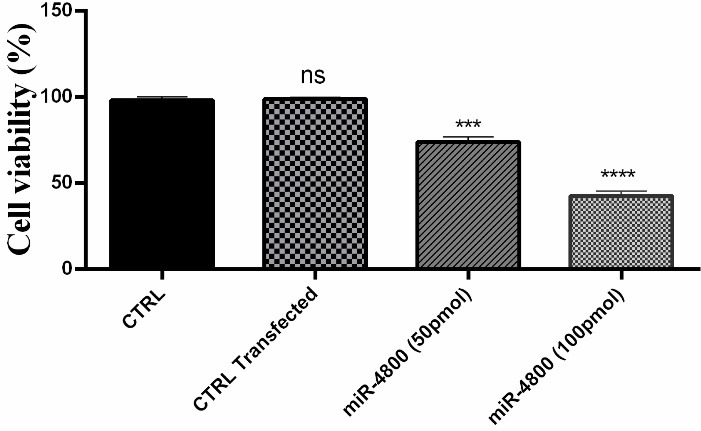
Effect of miR-4800 on breast cancer MDA-MB-231 cells viability. The data represent mean ± SD (n = 3) *** *P* < 0.001; **** *P* < 0.0001.

 The results of cell viability, which was studied by MTT assay as presented in Figure 2. These results indicated that cell viability rate was significantly decreased in the group transfected with the miR-4800 mimic when compared with the control group (*P* < 0.0001).

**Figure 3:**
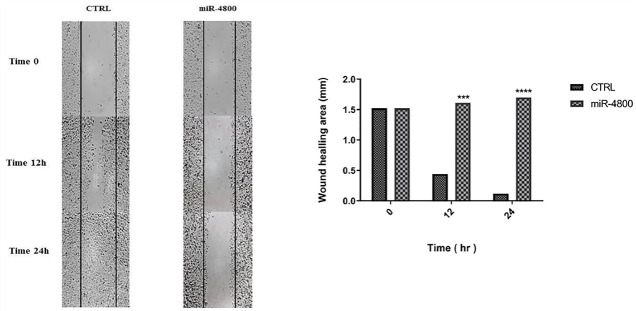
Effect of miR-4800 replacement on cell migration in breast cancer MDA-MB-231 cells by wound-healing assay. The replaced miRNA significantly decreased cell migration after 12 and 24 h. The data represent mean ± SD (n = 3), *** *P* < 0.001; **** *P* < 0.0001.

 The wound-healing assay was performed to evaluate the effect of miR-4800 replacement on cell migration of the MDA-MB-231 cell line (Figure 3). The present data indicated that the migration of BC cells was decreased after 12 (*P* < 0. 001) and 24 (*P* < 0.0001) hours of scratched time.

###  Replacement of miR-4800 decreased expression level of CD44, CXCR4, ROCK1, and Vimentin significantly

**Figure 4:**
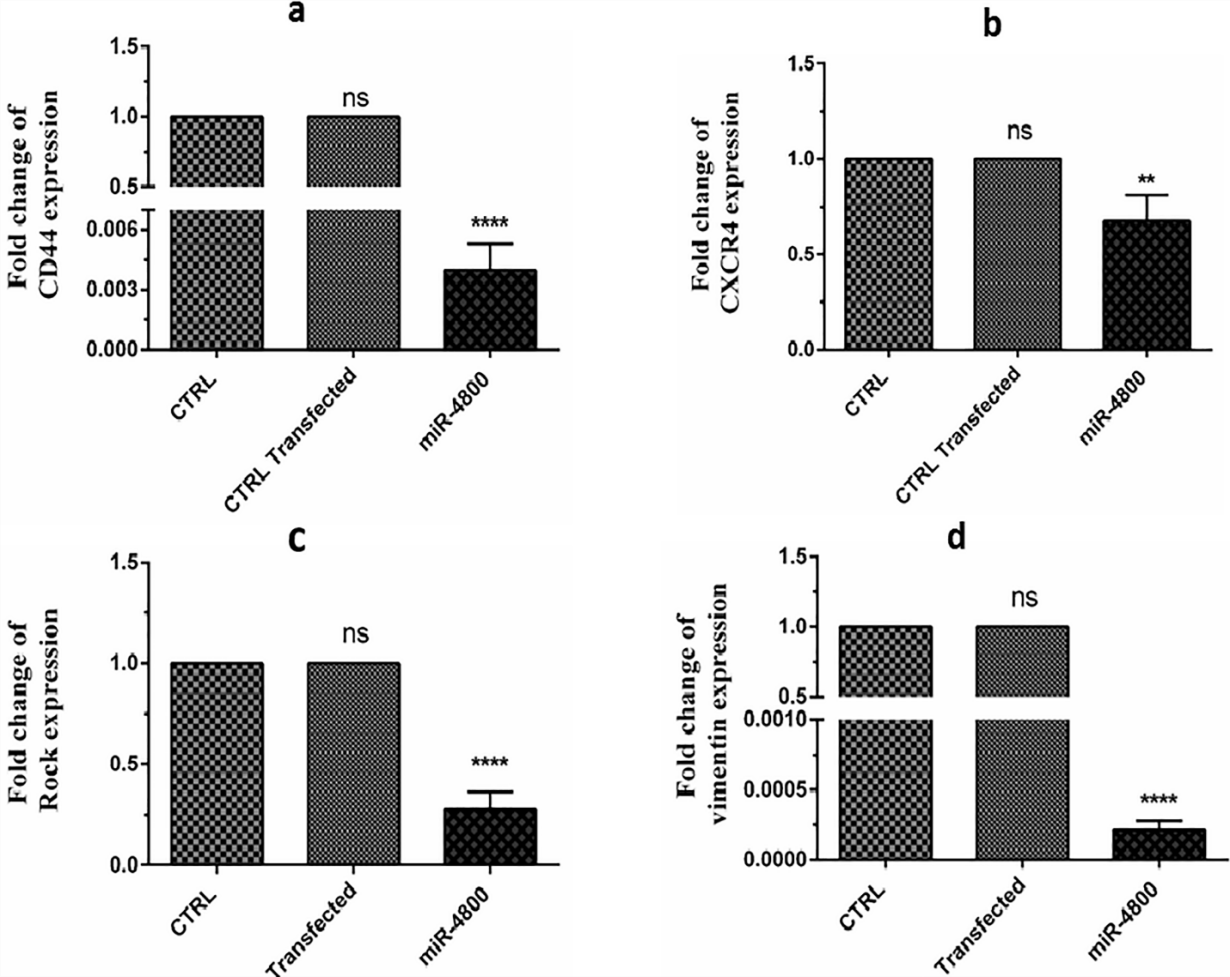
These data demonstrated the considerable decrement in the expression levels of CD44 (P < 0.0001), CXCR4 (*P* < 0.01), ROCK1 (*P* < 0.0001), and vimentin (*P*< 0.0001) genes compared to the control group. The data represent mean ± SD (n = 3).

 The expression levels of the selected genes after miR-4800 transfection are shown in Figure 4. The present findings determined that miR-4800 replacement resulted in down-regulation of CD44 (*P* < 0.0001), CXCR4 (*P* < 0.01), ROCK1 (*P* < 0.0001), and vimentin (*P* < 0.0001) when compared with the control cells.

###  Restoration of miR-4800 increased apoptosis in BC cells

**Figure 5:**
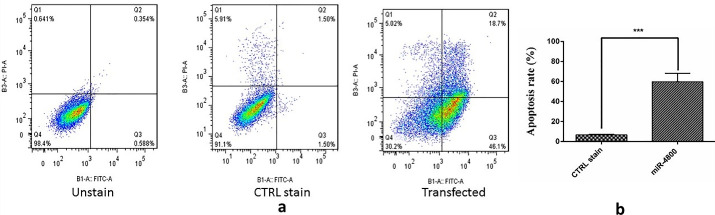
Effect of miR-4800 restoration on apoptosis induction in breast cancer MDA-MB-231 cells by flow cytometry assay (Annexin V/PI staining) which significantly decreased cell viability. The data represent mean ± SD (n = 3), **** P < 0.001. A diagram can be divided into four regions that are defined as follows: the percentage of necrotic cells (Q1: PI/FITC + /-); the percentage of late apoptotic cells (Q2: PI/FITC + / + ); the percentage of early apoptotic cells (Q3: PI/FITC -/ + ); the percentage of viable cells (Q4: PI/FITC -/-).

 Annexin V/PI assay was used to comprehend the impact of miR-4800 on apoptosis. The flow cytometry technique revealed remarkable differences in apoptosis rates after the replacement of miR-4800 (Figure 5) (*P* < 0.001). Interestingly, the percentage of early apoptosis (Q3 part in Figure) increased significantly (46.1%) in the transfected cells by miR-4800 when compared with other groups (0.588% and 1.50%). In the control group, the percentage of the viable cells was 91.1% (Q4).

## Discussion

 The miR-4800 family is located on chromosome4 (chr4: 2250077-2250156) (http://www.mirbase.org/), which has also been presented as an exonic miRNA and regulator of tumor suppressor expression.^18^ Several studies have been recently presented dysregulation (frequently downregulation) of miR-4800 in non-cancerous diseases and human cancers. However, little information is available on the biological roles of miR-4800 in cancer development. The current study is the first experimental design on the miR-4800 behavior in BC cells. The present data demonstrated the effect of miR-4800 transfection on BC cells, which subsequently decreased expression levels of some related genes involved in BC proliferation and migration, including ROCK1, CD44, CXCR4, and vimentin. Interestingly, restoration of miR-4800 could notably induce apoptosis in BC cells. On the other hand, MTT and scratch assays revealed significant inhibition of growth and cellular motility of BC cells. Thus, it was suggested that the miR-4800 transfection could attenuate proliferation, migration, and metastatic manner in BC cells *in vitro *by modulation of some related genes. However, little information is available on the biological roles of miR-4800-3p in cancer initiation, development, and progression. Based on the author҆ s knowledge, there is no experimental publication on the miR-4800 manner in cancer development. As previously described, deregulation of miR-4800 has been indicated in different cancers, such as BC,^7-9^ cervical cancer,^10^ colorectal cancer,^11,12^ ESCC, gastric cancer,^13^ glioma,^14^ liver carcinoma,^15,19^ and pancreatic cancer. Indeed, downregulation of miR-4800 was commonly investigated in patients with these cancers. Very recently, Pan et al­­­­^20^ decreased expression level of miR-4800 was investigated in CSF of the patients with lung adenocarcinoma. Moreover, miR-4800 represented a prognostic miRNA in hepatocellular carcinoma.^19^

 In the current study, we evaluated the expression levels of some genes that are commonly involved in BC proliferation and migration, including CXCR4, ROCK1, CD44, and vimentin after the restoration of miR-4800.

 ROCK1 is known as a substantial modulator of focal adhesion formation, cancer cell motility, and invasion.^21^ In this regard, growing evidence indicated the downregulation of ROCK1 by miR-193a and miR-340 in BC,^16,22^ miR-335 in osteosarcoma,^23^ miR-148a in gastric cancer,^24^ and miR-1280 in bladder cancer­^25^ could considerably suppress the cell migration, invasion, and metastasis manner of cancer cells. Based on the present findings. It seems that miR-4800 has a similar function in this line.

 CXCR4 (chemokine receptor type 4) overexpression is closely related to the increasing cancer migration behavior and metastasis phenotype in BC.^26^ In this way, previous studies revealed the impact of miR-145, miR-203,^27^ miR-302a,^28^ and miR-193a^16^ on the inhibition of invasiveness and metastatic feature of BC cells. The current data provided the same results upon miR-4800 restoration in migration of BC by scratch assay.

 Several studies demonstrated the interaction of CXCR4 regulation and EMT phenotype (a remarkable index of cellular plasticity) in BC with CD44 expression level. In this connection, it was reported that miR-520c and miR-373 enhance migration and invasion of cancer cells by targeting CD44 in BC.^29,30^ Besides, it was suggested that cancer cells that undergo EMT gain stem cell-like properties and represent CD44 overexpression.^31^ On the other hand, observation of EMT phenotype in cancer cells frequently leads to more resistance to chemotherapy.^32^ The involvement of CD44 in cancer progression provides that CD44 may be a suitable molecular target for cancer target therapy. Here, present findings suggest the downregulation of CD44 induced by the miR-4800 restoration may be associated with the expression level of CXCR4.

 Vimentin (a type III intermediate filament protein expressed in mesenchymal cells) has also been demonstrated as an indicator for pre-metastatic cancer cells undergoing EMT. Thus, its overexpression is related to worse outcomes in patients with solid cancer.^33^ In this way, several studies revealed the contribution of miR- 214,^34^ miR-138,^35^ miR-30a,^36^ and miR-193a^16^ to the EMT process in BC cells by vimentin expression. Of considerable interest, it is believed that overexpression of CD44 can upregulate the expression level of vimentin, which promotes the invasion and migration of cancer cells.^37^ Collectively, previous results are consistent with the current data, which provided the role of miR-4800 as an inhibitory miRNA for BC metastasis.

## Conclusion

 Alteration of miRNAs that act as an oncogene or a tumor suppressor can significantly contribute to the biology and progression of different cancers. In this regard, it seems that restoration of a tumor suppressor miRNA or silencing of an oncogene miRNA may control cancer development. Recently, dysregulation of miR-4800 has been reported in various cancer. The present data suggested that restoration of miR-4800 can reduce the malignancy of BC cells *in vitro*.

## Acknowledgments

 The authors are grateful to the Faculty of Veterinary Medicine, University of Tabriz, Tabriz, Iran, and also the Immunology Research Center, Tabriz University of Medical Sciences, Tabriz, Iran, for the financial support.

## Competing Interests

 The authors declare they have no conflict of interest.

## Ethical Approval

 Not applicable.
